# In renal cell carcinoma the PTEN splice variant PTEN-Δ shows similar function as the tumor suppressor PTEN itself

**DOI:** 10.1186/s12964-018-0247-9

**Published:** 2018-06-28

**Authors:** Ines Breuksch, Jonas Welter, Heide-Katharina Bauer, Thorsten Enklaar, Sebastian Frees, Joachim W. Thüroff, Annette Hasenburg, Dirk Prawitt, Walburgis Brenner

**Affiliations:** 1 Department of Gynecology, Johannes Gutenberg University Medical Center, Langenbeckstr. 1, 55131 Mainz, Germany; 2 Department of Urology, Johannes Gutenberg University Medical Center, Langenbeckstr. 1, 55131 Mainz, Germany; 3 Department of Pediatrics, Johannes Gutenberg University Medical Center, Langenbeckstr. 1, 55131 Mainz, Germany

**Keywords:** PTEN, Renal cell carcinoma, Splice variant, PTEN-Δ, Tumor progression, Metastasis

## Abstract

**Background:**

Loss of PTEN is involved in tumor progression of several tumor entities including renal cell carcinoma (RCC). During the translation process PTEN generates a number of splice variants, including PTEN-Δ. We analyzed the impact of PTEN-Δ in RCC progression.

**Methods:**

In specimens of RCC patients the expression of *PTEN-Δ* and *PTEN* was quantified. The PTEN expressing RCC cell line A498 and the PTEN deficient 786-O cell line were stably transfected with the *PTEN-Δ* or *PTEN* transcript. In Caki-1 cells that highly express PTEN-Δ, this isoform was knocked down by siRNA. Cell migration, adhesion, apoptosis and signaling pathways activities were consequently analyzed in vitro.

**Results:**

Patients with a higher *PTEN-Δ* expression had a longer lymph node metastasis free and overall survival. In RCC specimens, the *PTEN-Δ* expression correlated with the *PTEN* expression. PTEN-Δ as well as PTEN induced a reduced migration when using extracellular matrix (ECM) compounds as chemotaxins. This effect was confirmed by knockdown of *PTEN-Δ*, inducing an enhanced migration. Likewise a decreased adhesion on these ECM components could be shown in *PTEN-Δ* and *PTEN* transfected cells. The apoptosis rate was slightly increased by PTEN-Δ. In a phospho-kinase array and Western blot analyses a consequently reduced activity of AKT, p38 and JNK could be shown.

**Conclusions:**

We could show that the PTEN splice variant PTEN-Δ acts similar to PTEN in a tumor suppressive manner, suggesting synergistic effects of the two isoforms. The impact of PTEN-Δ in context of tumor progression should thus be taken into account when generating new therapeutic options targeting PTEN signaling in RCC.

**Electronic supplementary material:**

The online version of this article (10.1186/s12964-018-0247-9) contains supplementary material, which is available to authorized users.

## Background

**Fig. 1:**
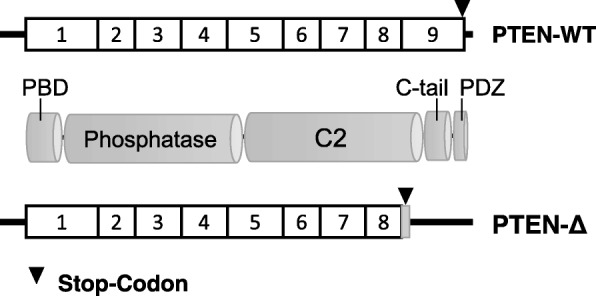
Schematic illustration of PTEN-Δ and PTEN. The nine exons of the *PTEN* gene are represented as boxes. *PTEN*-Δ lacks the coding potential of exon 9 due to inclusion of intron 8. The PTEN protein is composed of five domains: PBD-, Phosphatase, C2-, C-tail- and PDZ-binding Domain, PTEN-Δ consequently lacks parts of the C2 domain, the C-tail and the PDZ-binding domain

*PTEN* (Phosphatase and Tensin homolog on chromosome 10) encodes a tumor suppressor protein with dual specific protein and phospholipid phosphatase activity [1]. It is expressed ubiquitously and mediates cellular processes like adhesion, migration, cell survival and apoptosis [2]. The gene, located on chromosome 10q23.3, consists of 9 exons. The PTEN protein consists of 403 amino acids that are divided in five functional domains. From N-terminal to C-terminal PTEN includes a PBD-binding domain, a phosphatase domain, a C2 domain, a C-tail domain and a PDZ-binding domain (Fig. 1) [3]. The phosphatase domain comprises the catalytic center where the phosphatase dephosphorylates polypeptides or inositol rings [4]. The other domains take part in the subcellular localization and regulate the protein’s activity and degradation. Especially the C-terminal domains carry a lot of modification and protein-protein interaction sites [3].

In many tumor entities PTEN is inactive due to mutations, deletions or epigenetic changes [5–7]. The tumor suppressor is involved in several signaling pathways which can trigger tumor progression processes. By dephosphorylation of PtdIns(3,4,5)P_3_ (phosphatidylinositol 3,4,5-triphosphate) in the 3-position of the inositol ring [8], PTEN inhibits activation of the AKT signaling cascade and therefore reduces cell migration and adhesion and induces apoptosis [9–12]. Furthermore PTEN is able to dephosphorylate FAK (focal adhesion kinase) and SHC (SRC homology 2 domain containing) by its protein phosphatase activity. By doing so, PTEN regulates the interaction with the extracellular matrix (ECM), inhibits the MAPK (mitogen-activated protein kinase) pathway and hence modifies again cellular processes like proliferation, migration and adhesion [13, 14].

During mRNA processing PTEN generates a number of splice variants. Until now, ten different splice isoforms have been described [15, 16], eight of which include intron sequences in the open reading frame. PTEN-B, SV-5a, SV-5b and SV-5c contain parts of intron 5 and SV-3a, SV-3b and SV-3c contain parts of intron 3. The splice variant PTEN-Δ results from integration of intron 8 into the mRNA. In this splice variant the 61 amino acids of exon 9 are replaced by a valine and serine from intron 8, followed by a STOP codon (Fig. 1) [16]. Two other splice variants, SV-DelE5 and SV-DelE6, are generated by deletion of exon sequences. All splice variants lead to shorter protein sequences [15]. Agrawal and colleagues could show that SV-5c, SV-3b, SV-3c and SV-DelE6 are lower expressed in breast cancer specimens than in the corresponding healthy tissue. In contrast splice variant SV-5b showed a higher expression in the tumors [15]. However, the precise potential of the PTEN splice variants in tumor progression is still questionable and has to be determined in further studies.

In the presented study we analyzed the impact of the splice variant PTEN-Δ on tumor progression of renal cell carcinoma (RCC). We analyzed the *PTEN-Δ* expression values in RCC specimens and compared them with the survival rate and the status of metastasis. We demonstrate that *PTEN*-Δ is translated in RCC cell lines and analyzed the effect of *PTEN-Δ* overexpression or silencing on specific steps of tumor progression and metastasis in vitro.

## Methods

### Specimens

Primary RCC tissue samples were obtained under sterile conditions from 71 patients (Table 1) who underwent nephrectomy at the Department of Urology, University Medical Center Mainz [17]. The study was performed in agreement with the Declaration of Helsinki and approved by local ethics committee (No. 837.005.09, Landesärztekammer Rheinland-Pfalz, Mainz, Germany). Each patient provided informed consent. Samples of tumor tissue and renal cortex, obtained from the opposite kidney pole at a minimum distance of 3 cm from the tumor, were shock frozen in liquid nitrogen and stored at − 80 °C. The RCC diagnosis and tumor grade was verified on hematoxylin and eosin sections.

**Table 1:** Patient Data

		frequency
gender	male	49
	female	22
histology	clear cell	62
	papillary	9
pT-stage	1	23
	2	14
	3	34
	4	0
grading	1	3
	2	39
	3	25
	4	3
metastasis during follow-up	Yes	33
	No	34
	Unknown	4
age (years)	Median	61.33
follow-up (months)	Median	60.07
	Min	0.93
	Max	172.40

Overview of the composition of the analyzed patient cohort. The short minimal follow-up resulted from an early death of one patient

### RNA extraction

RNA was extracted from the frozen tissue samples by using the PeqGold Total RNA Kit (Peqlab). The tissue was first split into pieces of 40–50 mg and then transferred in Precellys ceramic tubes. Afterwards the samples were homogenized by adding 400 μl of RNA lysis buffer T (Peqlab) followed by incubation in a Precellys homogenizer on a frequence of 5000 rpm for 3 × 45 seconds. In contrast cultivated cell lines were lysed by adding 600 μl of RNA lysis buffer T and detached by using a scraper. Afterwards the samples were processed according to the kit-provider. To ensure a completely DNA free RNA extract an additional enzymatic digestion of DNA with DNase (Peqlab) was performed after the first washing step for 15 min at room temperature (RT). The amount of RNA in each sample was photometrically determined, using the Nanodrop1000 (Thermo Scientific).

### cDNA synthesis

RNA was transformed into cDNA with M-MLV reverse transcriptase (Invitrogen). Therefore 2–3 μg of isolated RNA was diluted in 20 μl RNase free water and denatured at 70 °C for 10 min. Next a reaction mix was added to every sample, each containing 1 μl oligo-dT primer (0.5 μg/μl), 2 μl RNase inhibitor, 3.5 μl dNTP mix (each 2.5 mM), 4 μl DTT (0.1 M), 8 μl of First strand buffer (0.1 M) and finally 1.5 μl M-MLV reverse transcriptase. Subsequently, the samples were incubated at 37 °C for 90 min and finally the reverse transcriptase was inactivated by raising the temperature on 95 °C for further 10 min.

### Real-time PCR

Quantification of gene expression was performed with the LightCycler 480 II (Roche). Specific primers were used to secure a selective amplification. These include the *PTEN*-specific primers 5‘-GTTTACCGGCAGCATCAAAT-3′ (forward) and 5‘-CCCCCACTTTAGTGCACAGT-3′ (reverse), as well as the *PTEN-∆* specific primers 5‘-TCCACAAACAGAACAAGATGC-3′ (forward) and 5‘-ACACACATCACATACATACAAG-3′ (reverse). The primers were added (10 μM each) to a total mixture of 10 μl, containing 5 μl Kapa SYBR Fast reagent (Kapa Biosystems), 3 μl distilled water and 1 μl of the cDNA sample. Each reaction was performed in duplicate and determined by the following program: initial denaturation (3 min; 95 °C), followed by 45 repetitive cycles, including denaturation (5 s; 95 °C), annealing (5 sec; 62–66 °C) and elongation (1 s; 72 °C). A final calculation of a melting curve concluded this analytic procedure. For analysis the geometrical average was calculated referring to the expression level of the house keeping genes TBP (TATA-box binding protein), ATP5J (ATP synthase, mitochondrial F0 complex subunit F6) and PPIA (peptidylprolyl isomerase A) [18], which were measured simultaneously.

### Cells and cell culture

The human RCC cell lines A498, 786-O, Caki-1 and Caki-2 were obtained from LGC Promochem and CCF-RCI and CCF-RCII were kindly provided by the establisher, Cleveland Clinic Foundation [19]. Caki-1 and Caki-2 cell culture was maintained in Iscoves medium (Biochrom), supplemented with 10% fetal calf serum, 1% GlutaMax (Sigma) and 1× penicillin/streptomycin (Life Technology). All other cell lines were maintained in RPMI1640 (Gibco) supplemented with 10% fetal calf serum, 2.5% HEPES buffer (Sigma) and 1× penicillin/streptomycin (Life Technology). All cell lines were incubated in a moistened atmosphere at 5% CO_2_ at 37 °C.

### Plasmid construction and stable transfection of RCC cell lines

ORFs of the *PTEN* and *PTEN-Δ* isoforms were PCR amplified, inserted into a pcDNA3 vector (Invitrogen) and in frame tagged with a V5 encoding domain. Chemocompetent *DH5 E. coli* cells were transfected with the expression plasmids. Individual clones were selected according to their resistance to ampicillin and verified by complete sequencing of the plasmid insert. For stable transfection 2 × 10^5^ A498 or 786-O cells were seeded in a 6-well-plate. Next day 5 μg plasmid DNA was mixed with 10 μl P3000 (Thermo Scientific) and 125 μl Opti-Mem (Gibco). At the same time 7.5 μl L3000 (Thermo Scientific) was diluted with 125 μl Opti-Mem. Both mixtures were combined and after 10 min incubation the cells were transfected by adding the mixture dropwise. After 24 h G418 (400 mg/ml) was added to the cell medium. Cells were cultured and resistant clones were selected.

### In vitro translation

For cell free in vitro translation (IVT) the 1-step human coupled IVT (Thermo Scientific) kit system was used. cDNA containing the ORF of the PTEN isoforms was inserted into the provided pT7CFE1 vector. For IVT 12.5 μl HeLa lysate was mixed with 2.5 μl accessory proteins. After incubation on ice for 10 min, 5 μl reaction mix, 2 μl of plasmid (0.5 μg/μl) and 3 μl nuclease free water were added. IVT was performed for 3 h at 30 °C.

### Knockdown

PTEN-∆ was selectively knocked down in Caki-1 cells, using the synthesized ds-siRNA targeting *PTEN-∆*: 5’-AAAUUUUAAGGUCAGUUAA-3‘ (sense) and 5’-UUAACUGACCUUAAAAUUU-3′ (antisense) from Sigma Aldrich. Lipofectamine RNAiMAX (Thermo Scientific) was used as transfection reagent. 2 × 10^5^ cells were plated in a 6-well-plate and cultured in 2.5 ml Iscoves medium lacking antibiotics. Each well received either *PTEN-∆* siRNA or scrambled locus siRNA (negative control) treatment as reference. In the following *PTEN-∆* siRNA, or scrambled locus siRNA and Lipofectamine RNAiMAX (7.5 μl) were each mixed with Opti-Mem complete medium (Gibco) to a total concentration of 150 pmol siRNA per well and in a total volume of 250 μl per well. The diluted transfection reagent was then mixed with the diluted siRNA solution and filled dropwise in the correspondent wells. After incubation at 5% CO_2_ and 37 °C for 24 h, medium was changed to Iscoves medium without additives and cells were incubated for another 24 h. Cells from each well were then detached, counted and prepared for a cell migration assay. The remaining cells of each sample were centrifuged, each suspended in 600 μl RNA lysis buffer T and frozen at − 80 °C for later RNA extraction.

### Cell adhesion assay

For cell adhesion assay amine-binding, maleic anhydride activated clear 96-well-plates (Pierce #15110, Thermo Scientific) were used as described previously [20]. Extracellular matrix components used were fibronectin (10 μg/ml), vitronectin (5 μg/ml), laminin (50 μg/ml), collagen I (10 μg/ml), collagen IV (10 μg/ml) and as control BSA (10 μg/ml) (all Thermo Scientific). Concentrations were selected according to the cell migration assay. Each well was coated with extracellular matrix components at a volume of 100 μl overnight on a rocking shaker at RT and washed twice with 100 μl washing buffer (DPBS with 0.05% Tween 20, ICI Amenic Inc.). Unspecific binding sites were blocked with 200 μl blocking solution (DPBS with 0.5% BSA) per well and incubated for 1 h in a moistened atmosphere at 5% CO_2_ at 37 °C in air. Meanwhile cells were washed in DPBS, medium was then replaced with Trypsin-EDTA and cells were resuspended in serum-free culture medium. Blocking solution was removed from the wells coated with ECM components and 50 μl of tumor cell suspension were added (4 × 10^5^ cells/ml) per well. After 1 h incubation in a moistened atmosphere at 5% CO_2_ at 37 °C in air, non-adherent cells were removed with 2 × 200 μl washing buffer (DPBS with 0.05% Tween 20) per well. Adherent cells were fixed with 100 μl 4% paraformaldehyde (Histofix 4%, Roth) for 15 min at RT and stained using 100 μl crystal violet solution (5 mg/ml in 2% ethanol) for 10 min at RT. Afterwards the staining solution was washed out with 3 × 100 μl washing buffer (DPBS with 0.05% Tween 20) per well and the plate was dried on air. For resolving the colorant wells were incubated with 100 μl 2% SDS (Roth) for 30 min. The adsorption was measured at 550 nm (reference value at 650 nm) with GloMax®-Multi detection system (Promega). Experiments were performed in quadruplicates and repeated three times.

### Chemotactic cell migration assay

For chemotactic cell migration analysis a modified Boyden chamber was used (Costar), as described previously [21]. The chamber consists of an upper and a lower department separated by a porous polycarbonate membrane with 8 μm pore diameter (Neuro Probe). Before analysis cells were cultivated in serum-free culture medium for 24 h. According to the instruction of the manufacturer, the lower chemotaxis compartment was filled with 29 μl solution of extracellular matrix components (according to the optimal conditions [22]: fibronectin 10 μg/ml, vitronectin 5 μg/ml, laminin 50 μg/ml, collagen I and IV 10 μg/ml (Thermo Scientific)) diluted in serum-free medium. The lower part was covered by the polycarbonate membrane, having been equilibrated in DPBS for 2 min. Then the upper part was fixed. The wells of the upper part were filled with 50 μl of tumor cell suspension (3 × 10^5^ cells/ml). The chamber was then incubated for 16 h in a moistened atmosphere with 5% CO_2_ at 37 °C. Afterwards not-migrated cells were removed from the upper membrane side by washing it in buffer solution according to Weise (Merck) and by mechanical detachment using a rubber scraper. Migrated cells were fixed in methanol for 1 min and dyed with hemacolor (Merck). The dyed membrane was transferred onto a microscope slide and covered with immersion oil. The migrated cells were counted on an area of 2.5 mm^2^ of the porous membrane. The experiment was performed in quadruplicates and repeated three times.

### Cell death detection assay

For apoptosis analysis in transfected RCC cells a cell death detection assay (Roche) was performed. 5 × 10^4^ cells were seeded in a 6-well-plate. Next day medium was discarded; cells were scraped off and transferred in 500 μl incubation buffer. After 30 min at RT a centrifugation at 20000 g for 10 min followed. The supernatant was used for cell death detection assay. For the assay the 96-well-plate was incubated with 100 μl coating solution per well over night at 4 °C. Next day the solution was discarded, 200 μl incubation buffer was added per well and incubated for 30 min at RT. A washing step with 300 μl washing solution per well followed for three times. 100 μl of the prepared supernatant was then filled in the wells. After 90 min the solutions were discarded and wells were washed again (3 × 300 μl/well washing solution). Incubation with 100 μl per well coating solution for 90 min at RT followed. After another washing step apoptosis was detected with 100 μl substrate solution per well. The adsorption of individual wells was measured at 405 nm with GloMax®-Multi detection system (Promega). Experiments were performed in quadruplicates and repeated three times. Mean value and standard error rate were calculated.

### Cell proliferation assay

To study the effect of transfected RCC cells on proliferation, a colorimetric BrdU incorporation assay (Roche) was performed as previously published [23]. The cells were seeded in quadruplicates into a 96-well-plate (5 × 10^3^ cells/well) and cultured for 48 h. BrdU solution (10 μM) was added to the cells and incubated for 2 h. The cells were fixed and the DNA was denatured in one step by adding 200 μl per well fixDenat solution for 30 min. Incorporated BrdU was detected by an anti-BrdU-POD antibody (100 μl antibody solution per well) within 60 min. Fixed cells were washed three times with 200 μl washing solution per well. The BrdU-antibody complex was detected by 100 μl substrate solution per well. After incubation of 15 min reaction was stopped by adding 25 μl H_2_SO_4_ (1 M) per well and proliferation was quantified by measuring the absorbance at 450 nm (reference 690 nm) with GloMax®-Multi detection system (Promega).

### Flow cytometric analysis of integrin subunits in renal carcinoma cell lines

The stably transfected 786-O and A498 cells were analyzed for the protein levels of integrin subunits α1, α2, α3, α5, αV, α6, β1 and β3. For this, cells were detached, centrifuged and washed with DPBS. Tumor cells (0.5 × 10^6^ cells) were resuspended in 100 μl DPBS + 1% BSA and treated with labeled anti-integrin subunit antibodies (integrin α1/CD49a PE-conjugated, integrin α3/CD49c Fluorescein-conjugated, integrin α6/CD49f Alexa Fluor 488-conjugated, integrin αV/CD51 PE-conjugated (all purchased from R&D Systems) and integrin α2/CD49b FITC-conjugated, integrin α5/CD49e PE-conjugated, integrin β1/CD29 PE-conjugated, integrin β3/CD61 FITC-conjugated (all purchased from Becton, Dickinson & Company) for 30 min on ice in darkness. Cells were washed by using DPBS and resuspended in 500 μl DPBS + 1% BSA for analysis. Fifteen-thousand counts were used for analysis (BD Calibur, Becton, Dickinson & Company). Protein expression analyses were performed three times.

### Human phospho-kinase array

The activity of 46 intracellular signaling kinases was quantified by using a human phospho-kinase array (R&D, Minneapolis, USA). The kinase array was performed according to the instructions in the manual. Protein extracts from RCC cells were prepared by using 200 μl lysis buffer 6 included in the kit. The cells were rinsed twice with ice-cold DPBS and scraped off with a cell scraper in lysis buffer. After 30 min incubation on ice, the extracts were centrifuged at 14000 g, 4 °C for 10 min. Protein concentrations were determined using BCA-reagents (Pierce BCA Protein assay kit, Thermo Scientific). The phospho-kinase array membranes were incubated with array buffer 1 for 1 h on a rocking platform. On each membrane 1 ml of the protein lysates (300 μg) was added and incubated overnight at 4 °C on a rocking platform. The membranes were washed three times with washing buffer and shaken with antibody cocktails for 2 h. After a 30-min treatment with streptavidin-HRP solution, the membranes were exposed to a chemiluminescent reagent. Signals were visualized using a chemiluminescent detector (FluorChemE, Protein Simple). For quantification a computer-based pixel counting system was used (AlphaView, Protein Simple).

### Western blot analysis

For preparation of protein extracts from cell culture, tumor cells (7.5 × 10^5^ cells) were seeded on 100 mm^2^ cell culture plates. For protein extraction, cells were washed with DPBS and mechanically detracted in lysis buffer (2 mM HEPES, 0.02 M NaCl, 0.05 mM MgCl_2_, 0.04 mM EDTA, 0.1% Triton X-100, 5 μM DTT, 1% Phosphatase Inhibitor Cocktail II (Sigma), 1% Protease Inhibitor Cocktail (Sigma)) with a cell scraper [24]. The solution was transferred into a 2 ml reaction tube and placed on ice. After incubation for 30 min on ice the samples were centrifuged for 10 min at 14000 g. The supernatant was transferred to a new tube and stored at − 20 °C. For evaluating protein concentrations of the extracts, BCA-reagents (Pierce BCA Protein assay kit, Thermo Scientific) were used. For protein precipitation 9-fold volume of acetone was used.

Equal amounts of protein (50 μg per lane) were separated by size using SDS-PAGE with 10% or 7.5% polyacrylamide gels. Afterwards gels were transferred on PVDF membrane by semi-dry blotting. Membranes were blocked according to instruction of antibody manufacturers for 1 h. Next, membranes were incubated with a primary antibody in blocking solution overnight at 4 °C on a roll mixer. The monoclonal antibodies against AKT, phospho-AKT S473 and T308, p38, phospho p38 (T180/Y182), PTEN (138G6), SAPK/JNK, phospho SAPK/JNK (T183/Y185) and V5-tag (all CST) were used at a dilution of 1:1000. β-actin antibody (Sigma) was employed at a dilution of 1:5000. After washing, the membranes were incubated with HRP-linked secondary antibodies (DAKO) at a dilution of 1:1000 for 1 h at RT and after washing bound antibodies were visualized by adding enhanced chemiluminescent solution (Perkin/Elmer) and detected in a chemiluminescent detector (FluorChemE, Protein Simple). For quantification bands were quantified by densitometry evaluation using a computer-based pixel counting system (AlphaView, Protein Simple). These values were referred to β-actin values of the same membrane as loading control. The cell culture experiments were performed three times.

### Statistical analysis

For statistical analysis we used IBM-SPSS 22.0 and Microsoft Excel 2013. Expression results were quantified and presented as relative units. Significances of tissue specimen analyses were calculated by using the Log Rank test in relation to lymph node metastasis and overall survival. Regression analyses were performed by using a Pearson correlation. All other results using RCC cell lines were presented in % of control cells. Differences in expression levels, activity levels, apoptosis rates, adhesion and migration potential were performed using the Student’s T-test. Statistical significance was assumed at a *p*-value of < 0.05.

## Results

### In the primary tumor of RCC patients *PTEN-Δ* expression correlates negatively with tumor progression

**Fig. 2:**
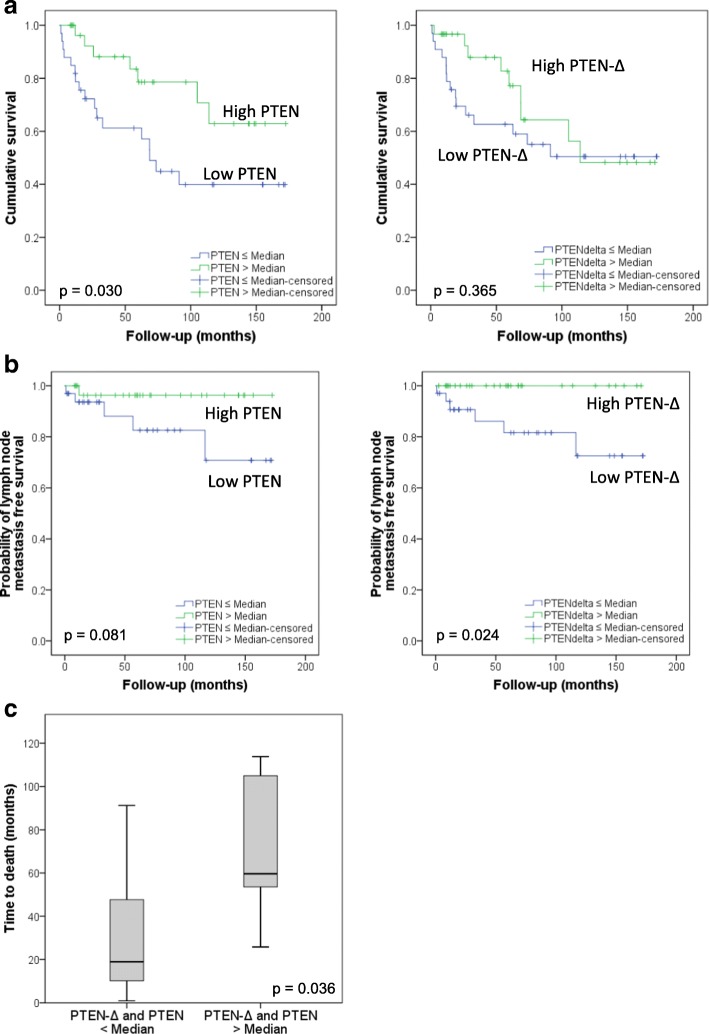
Influence of *PTEN-Δ* expression on tumor progression of RCC patients. *PTEN-Δ* and *PTEN* expression was quantified by Real-Time PCR in tumor tissue specimens from patients with primary RCC, shock frozen after nephrectomy. Patients were grouped in two categories: patients with a high *PTEN-Δ* or *PTEN* expression value (*PTEN-Δ* / *PTEN* > median) and in a group with low *PTEN-Δ* or *PTEN* expression value (*PTEN-Δ* / *PTEN* ≤ median). **a** The Kaplan-Meier curves show the lymph node metastasis rate of patients with high *PTEN-Δ* or *PTEN* expression value compared to patients with a low *PTEN-Δ* or *PTEN* expression in RCC tissue. Significance was calculated by a Log Rank test, *p* < 0.05. **b** The Kaplan-Meier curves show the overall survival of patients with high/low *PTEN-Δ* or *PTEN* expression value in RCC tissue. Significance was calculated by a Log Rank test, *p* < 0.05. **c** Comparison of time to death of patients with high *PTEN-Δ* and simultaneously high *PTEN* expression value (> median) and patients with low *PTEN-Δ* and simultaneously low *PTEN* expression (< median). Significance was calculated by a Mann-Whitney-U test, *p* < 0.05

The expression level of *PTEN-Δ* and *PTEN* was examined by Real-Time PCR in a cohort of 71 patients with RCC (Table 1). For analysis patients were distinguished in two groups, one with an expression value over median and one with an expression value less or equal to the median expression. Patients with a lower *PTEN-Δ* expression (*PTEN-Δ* ≤ median expression value) showed a significantly higher rate of lymph node metastasis than patients with a higher *PTEN-Δ* expression (*PTEN-Δ* > median expression value) (Fig. 2a, *p* = 0.024). For *PTEN* expression this relationship was not significant (Fig. 2a, *p* = 0.081). Concerning the *PTEN* expression value we could confirm its role as a tumor suppressor in our cohort, since patients with a high *PTEN* expression (*PTEN* > median value) had a better overall survival (Fig. 2b, *p* = 0.03). Furthermore, patients with a higher *PTEN-Δ* expression value tend to have a higher overall survival. In the first 5 years of follow-up the Kaplan-Meier curve of survival drifts between low and high *PTEN-Δ* expressing patients, but in the further course no significant difference was observed (Fig. 2b, *p* = 0.365). Patients who showed a high expression value in both *PTEN-Δ* and *PTEN* (> median) had a significantly longer survival compared to patients with low *PTEN-Δ* and simultaneously low *PTEN* expression value (< median) (Fig. 2c, *p* = 0.036). However, a combination of low *PTEN*/ *PTEN-Δ* expression or a combination of high *PTEN*/ *PTEN-Δ* expression was no independent predictor for survival or metastasis, as found by multivariate analyses (Cox regression), using histology, grading, staging and patient’s age for covariates (data not shown). The expression value of *PTEN* correlated with the expression level of its splice variant *PTEN-Δ*. While a low (≤ median) *PTEN-Δ* expression correlated with a low *PTEN* expression (35.21% of the patients), a high (> median) *PTEN-Δ* expression correlated with a high *PTEN* expression (35.21% of the patients). This correlation was significant (Table 2, *p* = 0.001).

**Table 2:** Cross table of *PTEN-Δ* and *PTEN* expression in tissue of 71 RCC patients

	*PTEN* ≤ median expression value	*PTEN* > median expression value
*PTEN-Δ* ≤ median expression value	25 (35.21%)	11 (15.49%)
*PTEN-Δ* > median expression value	10 (14.08%)	25 (35.21%)

The *PTEN* expression value correlates with the *PTEN-Δ* expression value. Significance was calculated by a Chi-Square test *p* < 0.05

### In RCC cells *PTEN-Δ* mRNA is translated into a PTEN-Δ protein

**Fig. 3:**
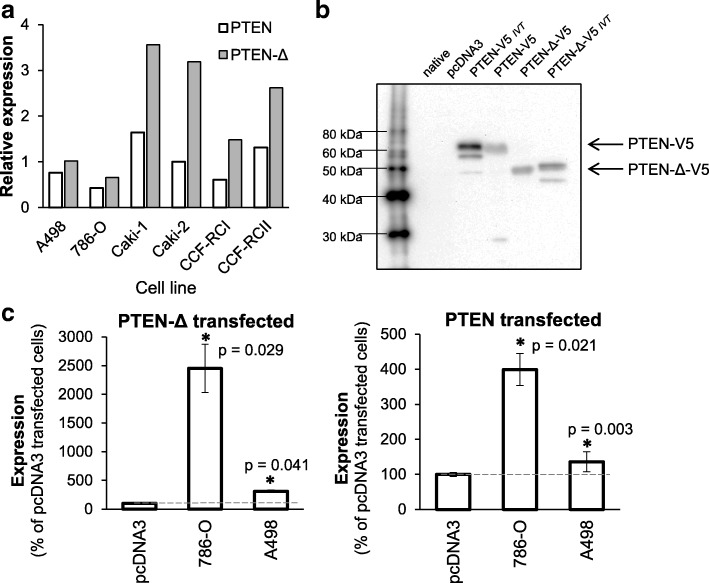
Expression of *PTEN-Δ* in RCC cell lines and translation of PTEN-Δ in RCC cells. **a** Relative expression values of *PTEN-Δ* in RCC cell lines were quantified by Real-Time PCR. Expression was normalized to expression of the house-keeping genes *TBP*, *ATP5J* and *PPIA*. **b** 786-O and A498 cells were transfected with the expression construct for the PTEN-Δ and PTEN isoform, C-terminally tagged with a V5-tag to enable detection of the PTEN-isoforms. Western blots of 786-O cell protein extracts were thus performed with an anti-V5-tag antibody. As positive control for the transfected constructs, cell free in vitro translated (IVT) protein of the respective construct was loaded. **c** Expression of *PTEN*-Δ and *PTEN* in transfected 786-O and A498 RCC cells were quantified by Real-Time PCR. Results show relative expression value of *PTEN-*Δ or *PTEN* compared to control cells (pcDNA3 transfected cells). Significance was calculated by Student‘s T-test, *p < 0.05

For analysis of the cellular function of PTEN-Δ we aimed to create PTEN-Δ overexpressing RCC cells, which endogenously express low level of PTEN-Δ. We screened the native expression of *PTEN-Δ* and *PTEN* by qRT-PCR in the RCC cell lines A498, 786-O, Caki-1, Caki-2, CCF-RCI and CCF-RCII. While Caki-1 and Caki-2 cells showed a high *PTEN-Δ* expression (3.56 and 3.19, relative units), in A498 and 786-O cells *PTEN-Δ* was relatively low expressed (1.02 and 0.66, relative units) (Fig. 3a). For determining the translation capability of *PTEN-Δ* we generated PTEN-Δ or PTEN in a cell-free in vitro translation (IVT), as a positive control. To study the influence of PTEN-Δ on tumor progression, we stably overexpressed PTEN-Δ and, for a comparison, PTEN in the PTEN-Δ low expressing RCC cell lines A498 and 786-O. A498 cells endogenously express a functional PTEN, the 786-O cells contain a mutation in the *PTEN* gene. As expected, Western blot analysis of these two cell lines verified that only A498 cells express endogenous PTEN on protein level (Additional file 1: Figure S1). After stable transfection, PTEN-Δ and PTEN were detectable on protein level in these cells, visualized by the use of an anti-V5-tag antibody (Fig. 3b), since no useable antibody for detection of PTEN-Δ could be found. The V5 tag-sequence was fused in frame to the 3′-end of the ORF of the *PTEN-Δ* and *PTEN* isoform, so that after translation it is located at the C-terminus of the protein. Overexpression of *PTEN-Δ* as well as *PTEN* was verified by quantitative RT-PCR with specific primers in both cell lines (Fig. 3c).

### PTEN-Δ reduces cell migration towards and adhesion to ECM components

**Fig. 4:**
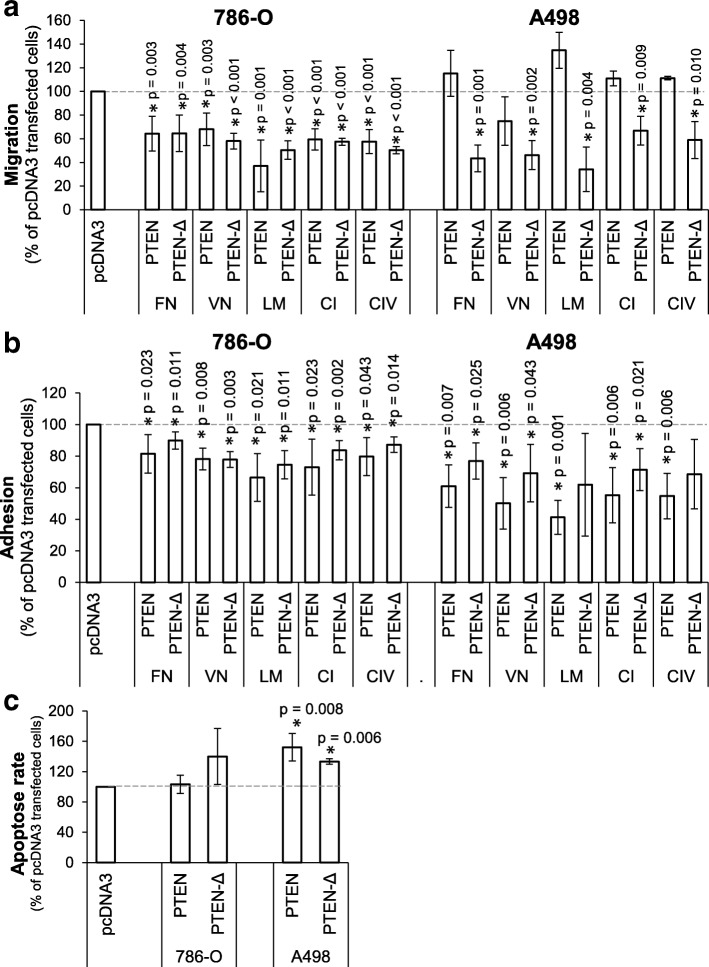
Influence of PTEN-Δ and PTEN on the functional behavior of renal tumor cells. **a** Migration of PTEN-Δ or PTEN transfected 786-O and A498 cells was determined in a Boyden chamber using ECM compounds as chemotaxins (FN: Fibronectin, VN: Vitronectin, LM: Laminin, CI: Collagen I and CIV: Collagen IV). Differences are shown in percentage of control cells (pcDNA3 transfected cells). **b** Cell adhesion of PTEN-Δ or PTEN transfected 786-O and A498 cells on immobilized ECM compounds were determined. BSA was used as control (Data not shown). Differences are shown in percentage of control cells (pcDNA3 transfected cells). **c** Apoptosis values of PTEN-Δ or PTEN transfected 786-O and A498 cells were quantified by determination of cytoplasmic histone-associated DNA fragments (Cell death detection assay, Roche). Differences are shown in percentage of control cells (pcDNA3 transfected cells). Significance was calculated by Student‘s T-test, *p < 0.05

We analyzed the effect of PTEN-Δ and PTEN on the chemotactical cell migration and cell adhesion in stably transfected 786-O and A498 cells. In 786-O cells overexpression of PTEN-Δ as well as PTEN resulted in a significant reduced migration using ECM components fibronectin, vitronectin, laminin, collagen I and IV as chemotaxins, compared to pcDNA3 vector transfected cells as control. The migration potential was decreased between 37 and 68% (Fig. 4a). In stably transfected A498 cells overexpression of *PTEN-Δ* induced a significant reduction in chemotactical cell migration between 34 and 67% of control, while *PTEN* overexpression had no effect (Fig. 4a). In addition to cell migration we analyzed the influence of PTEN-Δ on cell adhesion to the indicated ECM components. Both cell lines showed a decrease in cell adhesion in PTEN-Δ as well as in PTEN overexpressing cells. In 786-O cells the adhesion was reduced between 25 and 10% for PTEN-Δ and between 34 and 18% for PTEN (Fig. 4b). In A498 cells overexpression of PTEN-Δ showed a decrease between 61 and 76% comparing to pcDNA3 transfected cells. Overexpression of PTEN showed a similar effect with a reduced adhesion of 59 and 39% (Fig. 4b). In contrast, the proliferation was not influenced by either PTEN-Δ or PTEN overexpression (Additional file 2: Figure S2).

### The apoptosis rate is slightly triggered by PTEN-Δ

The apoptosis rate was determined by a cell death detection assay. In 786-O cells, PTEN-Δ triggered the apoptosis rate to 140% (not significant) comparing to pcDNA3 transfected control cells (Fig. 4c). In A498 cells overexpression of both PTEN-Δ and PTEN induced an enhanced apoptosis rate of 133% (*p* = 0.006) and 152% (*p* = 0.008), respectively (Fig. 4c).

### Knockdown of *PTEN-Δ* increases migration potential in Caki-1 cells

**Fig. 5:**
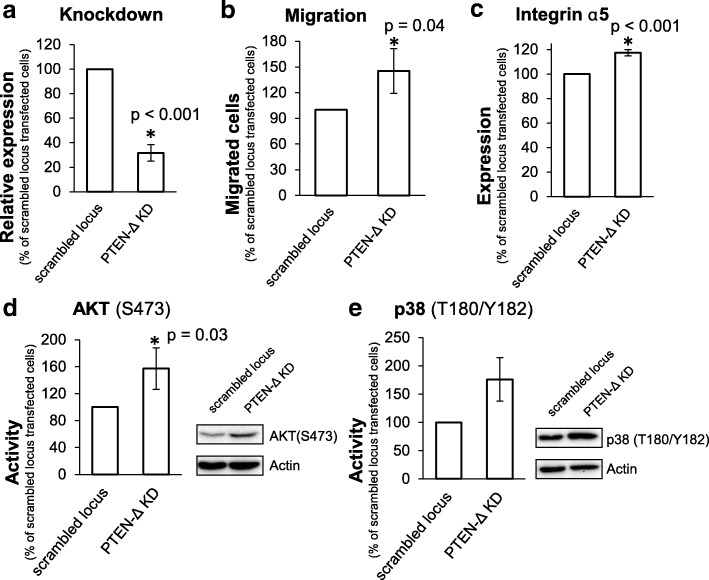
Verification of the role of PTEN-Δ. **a** The *PTEN-Δ* expression value of Caki-1 cells (native highly *PTEN-Δ* expressing) after *PTEN-Δ* siRNA knockdown (KD) was determined by Real-Time PCR. Expression was normalized to expression of the housekeeping genes *TBP*, *ATP5J* and *PPIA* and is shown as percentage of the expression of the nonsense siRNA transfection control (scrambled locus). Expression of *PTEN-Δ* was reduced to 32%. **b** Migration of Caki-1 cells after *PTEN-Δ* siRNA knockdown was determined in a Boyden chamber using fibronectin (10 μM) as chemotaxin. The migration value is shown as percentage of the migration of the scrambled locus siRNA transfection control. **c** Integrin α5 expression value of Caki-1 cells after *PTEN-Δ* knockdown was determined by flow cytometry. The expression value is shown as percentage of the transfection control (scrambled locus siRNA transfected negative control). Phosphorylation of AKT (S473) (**d**) and p38 (T180/Y182) (**e**) in Caki-1 cells after *PTEN-Δ* knockdown was determined by Western blot and quantified by densitometric evaluation of the bands. Results are shown as percentage of siRNA control transfected cells (scrambled locus). Significance was calculated by Student’s T-test, *p < 0.05

To verify the influence of PTEN-Δ on cellular processes, we exemplarily analyzed the effect of PTEN-Δ reduction on chemotactical migration in native *PTEN-Δ* expressing cells. Since Caki-1 RCC cells showed the highest expression of *PTEN-Δ* (Fig. 3a), we used these cells to study the effect of siRNA knockdown of *PTEN-Δ*. Knockdown efficiency of 68% (*p* < 0.001) was achieved, verified by qRT-PCR (Fig. 5a). We analyzed the influence of *PTEN-Δ* knockdown on chemotactical migration using fibronectin as chemotaxin. The *PTEN-Δ* knockdown increased the migration potential to 145% (*p* = 0.04), compared to scrambled locus (negative control) transfected cells (Fig. 5b).

### Integrin expression is influenced by PTEN-Δ

**Fig. 6:**
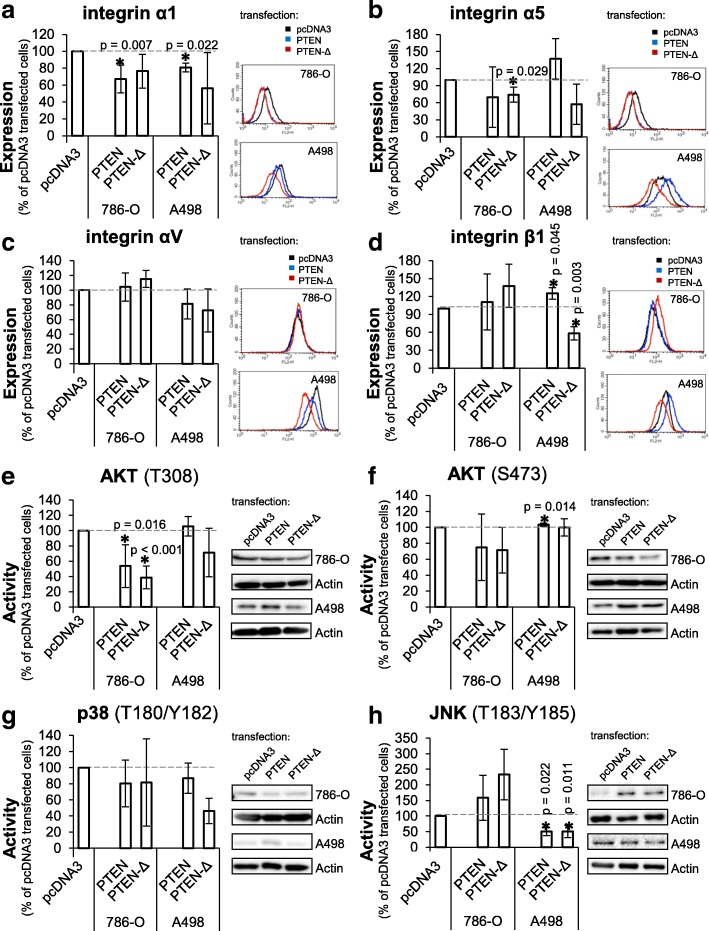
Influence of PTEN-Δ and PTEN on cell signaling. Expression of integrin α1 (**a**), integrin α5 (**b**) integrin αV (**c**) and integrin β1 (**d**) in RCC cells were determined in PTEN-Δ and PTEN transfected cells by flow cytometry. Representative histograms are shown for each staining. Expression value is shown as percentage of expression of the pcDNA3 transfected control cells. Significance was calculated by Student‘s T-test, *p < 0.05. Phosphorylation of AKT (T308) (**e**), AKT (S473) (**f**), p38 (T180/Y182) (**g**) and JNK (T183/Y185) (**h**) in transfected 786-O and A498 cells was determined by Western blot. Representative Western blots are shown for each kinase. Activity value was determined by densitometric evaluation and is shown as percentage of activity of the pcDNA3 transfected cells. Significance was calculated by Student‘s T-test, *p < 0.05

To examine whether the reduced migration and adhesion potentials are induced via an altered integrin expression, the integrin subunit expression of α1, α2, α3, α5, αV, α6, β1 and β3 were analyzed in the PTEN-Δ or PTEN overexpressing RCC cells. Both overexpressed isoforms reduced expression of integrin subunit α1 in 786-O cells (PTEN-Δ: 67.5%, not significant; PTEN: 67.2%, *p* = 0.007) as well as in A498 cells (PTEN-Δ: 56.2%, not significant; PTEN: 80.8%, *p* = 0.022) (Fig. 6a). Expression of integrin subunit α5 in 786-O cells was reduced to 74.2% (*p* = 0.029) in PTEN-Δ overexpressing cells and up to 70% (not significant) in PTEN overexpressing cells. In contrast in A498 cells, the integrin α5 expression was only in PTEN-Δ overexpressing cells reduced to 57.4% (not significant) but not in PTEN overexpressing cells (Fig. 6b). Integrin αV showed no effect in 786-O cells, but a decreased expression in PTEN-Δ and PTEN overexpressing A498 cells (not significant) (Fig. 6c). Integrin β1 expression was only decreased in PTEN-Δ overexpressing A498 cells (PTEN-Δ: 58.3%, *p* = 0.003) (Fig. 6d). All other integrin subunits tested were unchanged by PTEN-Δ or PTEN overexpression. These results demonstrate that PTEN-Δ, like PTEN, seems to regulate specific integrin subunit expression and inhibit migration and adhesion processes. Furthermore, we determined the integrin expression in Caki-1 cells after *PTEN*-Δ knockdown. Integrin α5 expression was slightly increased (+ 17%, *p* < 0.001) after knockdown of *PTEN*-Δ comparing to negative control (scrambled locus) (Fig. 5c).

### PTEN-Δ inhibits AKT and MAPK signaling cascade

Since PTEN-Δ inhibits cellular processes which participate in tumor progression, the involved signaling cascades were analyzed. We performed a human phospho-kinase array including 46 intracellular kinases (Additional file 3: Figure S3). The activities of the kinases were measured by detecting the expression of the phosphorylated molecules. Afterwards differences in activity levels exceeding 20% were verified by Western blot analysis. The activity of examined signaling molecules showed differences in the two RCC cell lines. In 786-O cells the strongest PTEN-Δ induced reduction was observed for phosphorylated AKT (T308) to 38.7% (*p* < 0.001), whereas PTEN reduced the T308 phosphorylation of AKT to 53.6% (*p* = 0.016) (Fig. 6e). Furthermore, in PTEN-Δ and PTEN overexpressing 786-O cells a moderate decrease of phosphorylated AKT (S473) and p38 kinase (T180/Y182) compared to pcDNA3 transfected cells was detected (Fig. 6f, g). In contrast, PTEN-Δ and PTEN overexpressing A498 cells showed a strong decrease of c-Jun N-terminal kinase (JNK) (T183/Y185) activity value (PTEN-Δ: 50.2%, *p* = 0.011; PTEN: 50.3%, *p* = 0.022) (Fig. 6h). In A498 cells the activity of the kinase p38 (T180/Y182) was also slightly reduced compared to control vector transfected cells (Fig. 6g). The kinase AKT showed a reduced phosphorylation on position T308 (Fig. 6e) only in PTEN-Δ overexpressing A498 cells. In addition, we determined the kinase activity in Caki-1 cells after *PTEN-Δ* knockdown. The kinase AKT showed an increased phosphorylation on S473 (+ 57%, *p* = 0.03) after knockdown of *PTEN-Δ* (Fig. 5d). The p38 kinase activity was also increased (to 176%) after *PTEN-Δ* knockdown compared to the transfection control (scrambled locus) (Fig. 5e). These results suggest a contribution of PTEN-Δ in regulation of both, AKT and MAPK, signaling cascades.

## Discussion

The tumor suppressor PTEN/MMAC1/TEP1 is known to be a regulator of cellular processes like cell proliferation, migration, motility and apoptosis [14, 25, 26], being frequently mutated in tumor cells. The cancer genome atlas identified more than 1120 mutations in 27 different tumor entities [27]. In RCC, several studies could show that PTEN shows a lower expression in tumor tissues compared to normal renal tissue and that patient’s with a higher expression in tumor tissue had a better outcome in context of survival and metastasis [5, 28, 29].

In this study we analyzed the impact of *PTEN’s* splice variant *PTEN-Δ* in tissues of patients with RCC. *PTEN-Δ* results from inclusion of intron 8 (Fig. 1). Consequently, the protein lacks the 61 amino acids of exon 9 which should be replaced by a valine and serine from intron 8 [16]. We demonstrate a putative contribution of the splice variant in tumor progression. Patients with high *PTEN-Δ* expression had a significantly lower risk of developing lymph node metastases. During the first 5 years of follow-up also patient’s cell death seems to occur faster when expressing *PTEN-Δ* low in tumor tissue. These results suggest that PTEN-Δ is likely to have a tumor suppressive role in RCC. Expression analyses of *PTEN* in our cohort confirmed its tumor suppressive role. Patients with a higher PTEN expression value had a significantly longer overall survival and tended to have a longer lymph node metastasis free survival. Patients with both, a high *PTEN*-Δ and a high *PTEN* expression value, had a significant longer time to death, indicating that both variants are important factors in regulating tumor processes. Furthermore, we analyzed the effect of PTEN-Δ on specific steps of tumor progression and metastasis in vitro. By using a V5-tag we could show for the first time that PTEN-Δ is translated and expressed on protein level in tumor cells. The splice variant lacks parts of the C2 domain and the C-terminal domains: the C-tail and the PDZ-binding domain (Fig. 1). These domains take part in PTEN’s stability and activity, since they contain many modification sites. By its PDZ-binding domain PTEN can interact with proteins containing also a PDZ domain, or with proteins that have a PtdIns(4,5)P_2_ motif. The membrane proteins MAGI, PAR-3, MAST, SAST and NEP, which contain such binding domains, are well-known examples of this fact. They can interact with PTEN and recruit the phosphatase to the membrane [30–32]. Localization of the protein to the membrane is also triggered via SUMOylation, whereas inhibition of this recruitment is regulated by phosphorylation of the C-tail, indicating that subcellular localization of PTEN is governed by a sensitive balance of modifications [33, 34]. Georgescu and colleagues could show that mutations in the C-terminal region lead to an instable, highly degradable protein [35]. In contrast, a deletion-mutant of PTEN shortened by 67 amino acids showed unchanged protein stability and functional properties [10]. These observations suggest that PTEN-Δ is stable and exerts phosphatase features in vitro.

Our functional analysis of important steps of tumor progression and metastasis showed that PTEN-Δ, like PTEN, acts in a tumor suppressive manner. PTEN-Δ overexpressing RCC cells had a lower migration potential towards ECM components, a reduced adhesion to these and showed an enhanced apoptosis rate. Analysis of the molecular setting showed a reduced expression of the integrin subunits α1, α5, αV and β1 as well as a decrease in JNK, p38 and AKT activity. Similar to the impact of PTEN-Δ on tumor progression in vivo*,* overexpression of PTEN in RCC cell lines 786-O and A498 also induced a reduced cell migration, reduced adhesion and enhanced apoptosis. These effects of PTEN were more intense in the cell line 786-O, which carries a mutation in PTEN gene (c.445C > T), than in A498 cells which already express PTEN endogenously. Probably A498 cells acquired a PTEN tolerance regarding tumorous effects and therefore are less affected by a PTEN overexpression. In glioma cells, Raftopoulou and colleagues could show that PTEN mediates cell migration processes via its C2 domain in a PI3K independent manner [25]. Further investigations demonstrated that PTEN intervenes in an integrin cascade, which triggered cell migration via tyrosine kinases of the SRC family [36]. Comparably we could show in former studies that PTEN regulates migration processes in 786-O cells via its protein phosphatase activity in a SHC dependent signaling cascade [10]. Here, we could demonstrate that PTEN-Δ as well as PTEN overexpression leads to a decrease in integrin subunit α1, α5 and αV expression, and that also the MAPK’s JNK and p38 showed a reduced activity value. Furthermore, we could show that a knockdown of PTEN-Δ lead to an increase in integrin α5 expression and a higher activity of the kinases AKT and p38. While β1 integrin together with α1 forms a heterodimer receptor for laminin and collagens, an α5/β1 complex forms a receptor for fibronectin and an αV/β1 dimer for vitronectin [37]. Corresponding to the reduced integrin expression the migration in direction and adhesion to these ECM components was reduced. This data suggests that PTEN-Δ inhibits cell migration and adhesion via integrin and MAPK signaling cascade similar to PTEN. A contribution of JNK in the MAPK cascade to PTEN regulation has already been shown by Vivanco and colleagues [38]. The results indicate that PTEN-Δ and PTEN possibly regulate migration and adhesion processes via a changed integrin subunit expression and downstream via the MAPK signaling cascade. On the other hand it is also possible that the reduced activity of MAPK signaling cascade induces the decreased integrin expression and subsequently leads to the reduced cellular processes. The PI3K is the main antagonist of PTEN, which dephosphorylates PtdIns(3,4,5)P_3_ signaling messengers and thereby inhibits downstream AKT activation [2]. We can observe a decrease in AKT activity in PTEN-Δ and PTEN overexpressing 786-O cells. Like in Schneider and colleagues in RCC cells [10], we can confirm a contribution of PTEN in this regulation. We show that also PTEN-Δ is capable to reduce AKT phosphorylation and therefore can inhibit this signaling cascade. Cellular signaling processes downstream of AKT, like cell proliferation, migration and apoptosis, get consequently modified. Cell proliferation was neither influenced in PTEN-Δ nor PTEN overexpressing RCC cells (Additional file 2: Figure S2) although it has been described that PTEN is able to regulate these processes [39]. This indicates that in the used RCC cells proliferation is regulated via different signaling cascades.

**Fig. 7:**
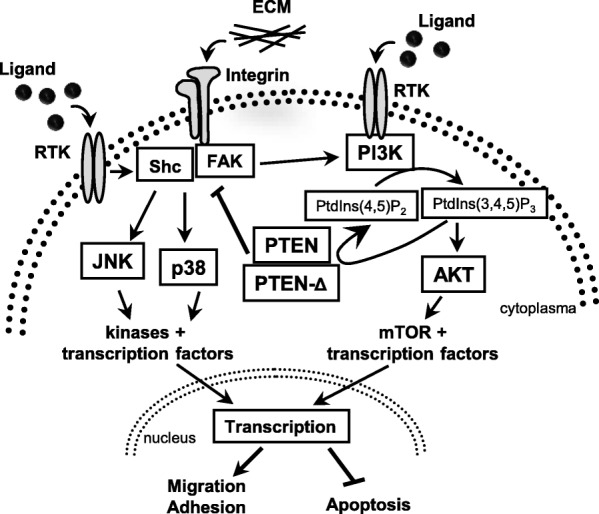
Schematic illustration of the impact of PTEN-Δ and PTEN on cellular processes. PTEN-Δ, as well as PTEN, inhibit AKT as antagonist of the PI3K and reduce the activity of MAPK JNK and p38. Both signaling pathways downstream result in a decrease of migration and adhesion and trigger apoptosis

A498 cells endogenously have a functional PTEN, while in 786-O cells the PTEN gene bears a mutation. In both cell lines a similar impact of *PTEN*-Δ overexpression on the analyzed cellular processes could be observed. Effects in A498 cells are more intense compared to 786-O cells, arguing for some kind of synergistic effect of both isoforms probably through interactions or dimerization of the isoforms, a function which was recently shown by Papa and colleagues [40]. Our results indicate that PTEN-Δ acts as a new tumor suppressor for RCC. An artificial effect of the synthetic PTEN-Δ overexpression is unlikely, since knockdown of *PTEN*-Δ in Caki-1 cells confirmed our finding by leading to a drop of migration potential when using fibronectin as chemotaxin. The splice variant acts probably in a similar fashion as the full length protein PTEN via the integrin and MAPK cascade and by inhibiting the AKT pathway. In this way it may diminish and finetune cellular processes like migration, adhesion and prevention of apoptosis (Fig. 7).

## Conclusions

The diversity of the tumor suppressor protein PTEN is substantiated by its diverse splice variants. These splice variants might also take part in tumor progression processes. Our data demonstrates that PTEN-Δ acts similar to PTEN in a tumor suppressive manner. Knowledge about the detailed individual function of PTEN-Δ and other splice variants of PTEN in the context of tumor progression might uncover new therapeutic options in RCC and other tumor entities.

## Additional files

Additional file 1: **Figure S1.** Western blot of A498 and 786-O cells. Protein extracts of A498 and 786-O cells were analyzed concerning the expression value of PTEN. (PDF 89 kb)

Additional file 2: **Figure S2.** Influence of PTEN-∆ and PTEN on proliferation. Proliferation was determined by BrdU incorporation. Differences are shown as percentage of the transfection control cells (pcDNA3 transfected cells). (PDF 90 kb)

Additional file 3: **Figure S3.** Human phospho-kinase array (Roche) of transfected 786-O and A498 cells. Protein extracts were obtained from PTEN-Δ and PTEN transfected cells and analyzed concerning the phosphorylation status of 46 intracellular signaling kinases. The activity of the kinases AKT, JNK and p38 are highlighted with red boxes. (PDF 111 kb)

## Abbreviations

AKT: AKT8 virus oncogene cellular homolog
ATP5J: ATP synthase, mitochondrial F0 complex subunit F6
ECM: Extracellular matrix
ERK: Extracellular signal-regulated kinase
FAK: Focal adhesion kinase
IVT: In vitro translation
JNK: c-Jun N-terminal kinase
MAGI: Membrane-associated guanylate kinase
MAPK: Mitogen-activated protein kinase
NEP: Nuclear export protein
PI3K: Phosphatidylinositol 4,5-bisphosphate 3-kinase
PPIA: Peptidylprolyl isomerase A
PtdIns(3,4,5)P_3_: Phosphatidylinositol 3,4,5-triphosphate
PtdIns(4,5)P_2_: Phosphatidylinositol 4,5-diphosphate
PTEN: Phosphatase and tensin homolog on chromosome 10
qRT-PCR: quantitative Real-Time PCR
RCC: Renal cell carcinoma
RT: Room temperature
RTK: Receptor tyrosine kinase
SAST: Syntrophin-associated serine/threonine kinase
SDS-PAGE: Sodium dodecyl sulfate polyacrylamide gel electrophoresis
SFK: SRC-family kinase
SHC: SRC homology 2 containing
SRC: Rous sarcoma oncogene homologue
SV: Splice variant
TBP: TATA-box binding protein
