# Supplementary figures and images for: In renal cell carcinoma the PTEN splice variant PTEN-Δ shows similar function as the tumor suppressor PTEN itself
Source: Cell Commun Signal. 2018 Jun 28;16:35. doi: 10.1186/s12964-018-0247-9 (PMC6025732; doi:10.1186/s12964-018-0247-9)

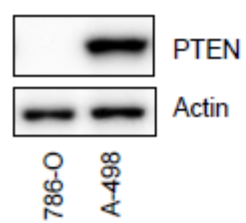

supplementary figure 1

Supplement: Supplementary file 1 — Figure S1. Western blot of A498 and 786-O cells. Protein extracts of A498 and 786-O cells were analyzed concerning the expression value of PTEN. (PDF 89 kb) [file 12964_2018_247_MOESM1_ESM.pdf]

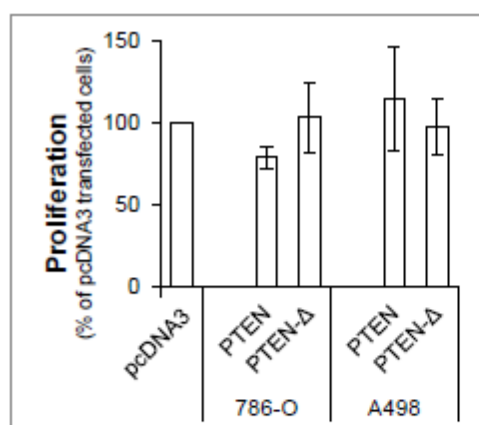

supplementary figure 2

Supplement: Supplementary file 2 — Figure S2. Influence of PTEN-∆ and PTEN on proliferation. Proliferation was determined by BrdU incorporation. Differences are shown as percentage of the transfection control cells (pcDNA3 transfected cells). (PDF 90 kb) [file 12964_2018_247_MOESM2_ESM.pdf]

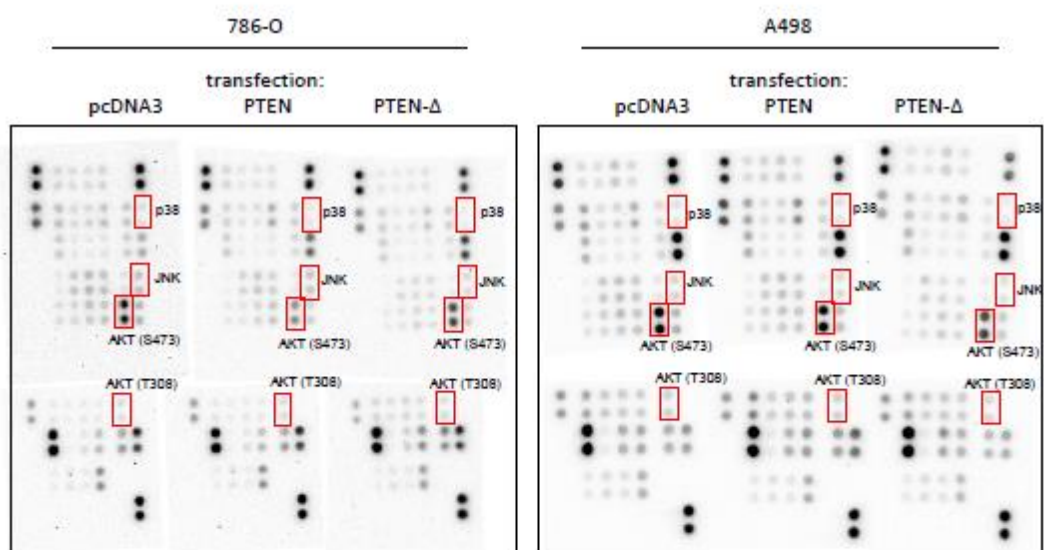

supplementary figure 3

Supplement: Supplementary file 3 — Figure S3. Human phospho-kinase array (Roche) of transfected 786-O and A498 cells. Protein extracts were obtained from PTEN-Δ and PTEN transfected cells and analyzed concerning the phosphorylation status of 46 intracellular signaling kinases. The activity of the kinases AKT, JNK and p38 are highlighted with red boxes. (PDF 111 kb) [file 12964_2018_247_MOESM3_ESM.pdf]
